# Skeletal Tuberculosis: Clinical and Radiological Profile, Management, and Outcomes in a Retrospective Study

**DOI:** 10.7759/cureus.97948

**Published:** 2025-11-27

**Authors:** Vinay Kumar SI, Akash Hosthota, Vinutha D, Bharath HD, Harish S Pai

**Affiliations:** 1 Orthopedics, Subbaiah Institute of Medical Sciences, Shivamogga, IND; 2 Orthopedics and Spine Surgery, Subbaiah Institute of Medical Sciences, Shivamogga, IND; 3 School of Medicine, Subbaiah Institute of Medical Sciences, Shivamogga, IND

**Keywords:** elbow tuberculosis, extrapulmonary tuberculosis, hip tuberculosis, knee tuberculosis, retrospective study, shoulder tuberculosis, skeletal tuberculosis, spinal tuberculosis, sternal tuberculosis, wrist tuberculosis

## Abstract

Introduction

Skeletal tuberculosis (TB) is a diagnostically challenging form of extrapulmonary TB (EPTB). Among EPTB, musculoskeletal or osteoarticular tuberculosis (OAT) represents 1-3% of all TB cases. This study evaluated the clinical patterns, diagnostic pathways, and treatment outcomes of skeletal TB cases managed under the National Tuberculosis Elimination Program (NTEP).

Methods

A retrospective review was conducted at the Subbaiah Institute of Medical Sciences, a tertiary-care center in Karnataka, India, including 20 patients with skeletal TB treated under NTEP between January 2022 and January 2025. Inclusion required completion of NTEP-based anti-tubercular therapy (ATT) and ≥1-year post-treatment follow-up. Diagnosis followed the NTEP criteria using an integrated approach involving clinical features, MRI, histopathology demonstrating necrotizing granulomatous inflammation, and microbiological testing (GeneXpert MTB/RIF). Functional outcomes were assessed using the Visual Analog Scale (VAS) for pain, Frankel grading for spinal cases, and validated joint-specific scoring systems (Knee Society Score, Modified Harris Hip Score, Mayo Elbow Performance Score, QuickDASH, Constant-Murley Shoulder Score) in extraspinal cases. Treatment outcomes were evaluated according to NTEP-defined clinico-radiological criteria.

Results

Spinal TB was observed in 14 patients (70%), predominantly lumbar (6/14), and extraspinal involvement in six patients (30%), affecting the hip, knee, shoulder, elbow, wrist, and sternum. All patients underwent MRI and biopsy; GeneXpert detected no rifampicin resistance. Surgical intervention was performed in cases with neurological deficits, instability, large abscesses, or advanced joint destruction. Mean VAS pain scores improved significantly from 7.35 ± 1.23 pre-treatment to 2.40 ± 0.94 at one year (p < 0.001). Functional scores in extraspinal cases ranged from fair to excellent across joints. Neurological status remained grade E in all spinal cases. All patients achieved treatment completion with clinico-radiological cure, including those with comorbidities and HIV infection.

Conclusion

NTEP-guided composite diagnosis, combined with selective surgery and standardized ATT, resulted in significant pain improvement and favorable outcomes in skeletal TB. Early biopsy and MRI are crucial for timely diagnosis and reducing disability.

## Introduction

Tuberculosis (TB) continues to be a significant global health challenge. According to the 2024 World Health Organization (WHO) report, approximately 10.8 million new cases and nearly 1.25 million deaths were reported worldwide [1]. While pulmonary TB accounts for the majority of cases (≈85%), extrapulmonary TB (EPTB) contributes to around 15% of the total disease burden [2]. Within EPTB, musculoskeletal or osteoarticular tuberculosis (OAT) represents a small but clinically significant subset, occurring in 1-3% of all TB cases [2].

Skeletal TB comprises 5-20% of all TB infections, with spinal involvement being the most common presentation, accounting for approximately half of these cases [1,3]. Spinal TB, or Pott’s disease, predominantly affects the thoracic and lumbar vertebrae [4]. Its clinical manifestations are insidious and nonspecific, frequently resulting in diagnostic delays of four to six months [5]. Such delays often contribute to complications, including neurological impairment, spinal deformity, and poor functional outcomes, with many patients never regaining full neurological recovery [6,7]. Early initiation of anti-tubercular therapy (ATT), however, achieves cure rates as high as 95% in early-stage disease [8], highlighting the critical role of timely diagnosis.

Beyond the spine, the knee, hip, and ankle are the most frequently affected joints [9,10]. Hip TB constitutes approximately 15% of OAT cases, typically presenting with gradually progressive hip pain, restricted motion, and eventual deformity [11-13]. In the upper limb, elbow TB, although rare, accounts for 1-5% of OAT and has been reported as the second most common site after the knee in some cohorts [10,14]. Other uncommon manifestations include tuberculous tenosynovitis, most often involving the wrist and hand flexor tendons, representing around 5% of OAT [15], and wrist joint TB itself, which is extremely rare (1-2%) but associated with significant morbidity due to loss of hand function [16,17]. Shoulder involvement is even less common, accounting for only 1% of cases [18,19]. The diverse clinical spectrum of musculoskeletal TB has earned it the description of a “great mimicker,” owing to its nonspecific presentations and ability to resemble various infectious and noninfectious conditions on both clinical and radiological grounds [20]. This frequently leads to delayed diagnosis and inappropriate initial management. Recent evidence across global TB analyses, narrative reviews of spinal TB, and contemporary case-based literature consistently indicates that diagnostic delays, frequent misclassification as degenerative or malignant spinal disorders, and limited access to timely microbiological confirmation remain major challenges in the diagnosis and management of musculoskeletal TB [1,5,6]. Greater awareness of its varied manifestations, particularly in endemic regions, is essential to enable early recognition, rational use of advanced imaging and biopsy, timely initiation of therapy, and judicious selection of surgical intervention.

The objectives of this study were to describe the clinical and radiological profile of patients with skeletal TB managed at a tertiary care center; to outline the diagnostic pathways using MRI, histopathology, and microbiological testing in accordance with National Tuberculosis Elimination Programme (NTEP) guidelines; and to evaluate treatment outcomes (medical ± surgical) using validated scoring systems at the ≥1-year post-treatment follow-up after completion of NTEP-based therapy.

## Materials and methods

Study design and ethical approval

This retrospective study was conducted at the Subbaiah Institute of Medical Sciences, a tertiary care hospital in Shivamogga, Karnataka, India. After obtaining approval from the Institutional Ethics Committee (IEC-SUIMS/148), data collection was initiated. The study reviewed skeletal TB cases registered and treated under the National Tuberculosis Elimination Program (NTEP) between January 2022 and January 2025. Patients were eligible only if they had completed NTEP-based ATT and had at least one year of follow-up after completion of treatment. As this study was retrospective, direct physical reassessment was not feasible; therefore, telephonic contact was attempted, and verbal consent was obtained. The study adhered to the ethical principles of the Declaration of Helsinki and followed NTEP diagnostic and treatment guidelines.

Case identification and selection

A priori sample size calculation was not possible because this was a retrospective case-series study based on the number of available eligible cases. Twenty skeletal TB cases met the inclusion criteria over the three-year period. Case retrieval was performed using multiple institutional sources, including the NTEP TB treatment register, electronic medical records searched using ICD-10 codes A17 and M49, operation theatre records documenting decompression, stabilization or synovectomy procedures, and histopathology reports describing granulomatous inflammation compatible with TB. Demographic information, anatomical site of involvement, clinical presentation, diagnostic modalities utilized, treatment protocol administered, and follow-up outcomes were extracted from these records.

Inclusion and exclusion criteria

Patients were included if they fulfilled the NTEP diagnostic criteria for skeletal TB, presenting with clinical features suggestive of musculoskeletal TB, imaging findings consistent with bone or joint involvement, and at least one supportive finding - necrotizing granulomatous inflammation on histopathology, microbiological confirmation via GeneXpert MTB/RIF, AFB smear or culture, or a sustained clinicoradiological response to ATT when microbiological confirmation is not possible. Only patients registered under NTEP who completed the full course of ATT and had a minimum of one year of documented post-treatment follow-up with adequate clinical and radiological records were included.

Patients were excluded if alternative diagnoses such as pyogenic osteomyelitis or malignancy were established by histopathology or microbiology, if ATT was not completed or was discontinued, if post-treatment follow-up was less than one year, or if clinical or radiological records were incomplete and did not allow reliable data extraction.

Imaging

Magnetic resonance imaging (MRI) was the primary imaging modality used in all patients. Examinations were performed on a 1.5-Tesla scanner using T1-weighted, T2-weighted, short tau inversion recovery (STIR) sequences and contrast-enhanced fat-suppressed T1 images. Radiological features evaluated included paradiscal erosion, marrow edema, cortical destruction, paravertebral or periarticular abscess formation, narrowing or destruction of joint spaces, synovial hypertrophy, and neural compression in spinal cases. Reporting was carried out by board-certified radiologists, and complex cases underwent multidisciplinary review involving orthopaedic surgeons and radiologists to minimize interpretation bias.

Histopathology and microbiology

Biopsy specimens were obtained either through CT-guided core needle biopsy or open surgical biopsy, including synovectomy for affected joints. Microscopic examination revealed necrotizing granulomatous inflammation with or without Langhans giant cells in all patients and was interpreted in conjunction with clinical and radiological findings. GeneXpert MTB/RIF testing was performed in all cases.

Treatment protocol

All patients received treatment according to NTEP weight-band dosing. The regimen included an intensive phase of two months consisting of isoniazid, rifampicin, pyrazinamide, and ethambutol (HRZE), followed by a continuation phase of isoniazid, rifampicin, and ethambutol (HRE) for 10 months. Surgical management was reserved for patients presenting with neurological deficit, spinal instability, large cold abscesses requiring drainage, or advanced joint destruction affecting function.

Outcome assessment

Outcome was defined according to the NTEP as treatment completion with clinico-radiological improvement and without the need for reinstatement of therapy or further surgical intervention for ≥1 year after ATT completion. The assessment of treatment outcome was based on NTEP-defined clinico-radiological criteria, which are recommended for extrapulmonary TB due to the frequent absence of follow-up microbiological confirmatory tests.

Pain and functional outcomes were assessed at baseline and at the end of the one-year post-treatment follow-up using Visual Analog Scale (VAS) pain scores, Frankel grading for neurological status in spinal cases. In extraspinal cases, joint-specific functional outcomes were assessed using validated scoring systems, including the Knee Society Score, Modified Harris Hip Score, Mayo Elbow Performance Score, QuickDASH (wrist), and the Constant-Murley Shoulder Score. Scores were not assessed at ATT completion as functional and neurological recovery continue beyond drug cessation and stabilize over the year after completion.

Statistical analysis

Continuous variables were summarized using mean values with standard deviations or medians with interquartile ranges, depending on distribution. Categorical variables were expressed as frequencies and percentages. As the data were non-normally distributed and the sample size was limited, pre- and post-treatment VAS scores were compared using the Wilcoxon signed-rank test. A p-value of less than 0.05 was considered statistically significant.

## Results

**Figure 1:**
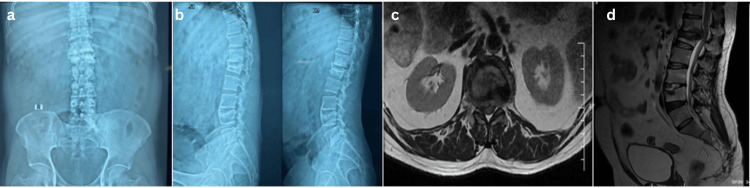
Spinal case 4 (a) Plain X-ray of the lumbar spine in the anteroposterior (AP) view shows the collapse of the L1 vertebrae with paradiscal sclerosis at D12 and the L1 disc level. (b) Plain X-ray of the lumbar spine (flexion and extension) in the lateral view shows compression of L1 with loss of height and no significant movement on dynamic views. (c, d) MRI of the lumbar spine in the axial view and T2 sagittal views, respectively, showing severe T12-L1 disc retropulsion with compression of the conus medullaris and nerve roots, along with reactive inflammatory changes at this level. The data shown are representative radiographic and MRI findings from a single patient case (spinal case 4). Original images obtained by the authors at Subbaiah Institute of Medical Sciences, Shivamogga.

In this retrospective series of 20 patients, demographic details (age and sex), anatomical sites involved, clinical history, diagnostic modalities used, clinical presentations, the treatment regimens administered, and the outcome of spinal cases are tabulated in Table 1, and those of the extra-spinal cases are in Table 2. Representative radiographic and MRI findings of lumbar spinal tuberculosis are demonstrated in Figure 1.

**Table 1:** Clinical, radiological, and treatment profile of patients with spinal tuberculosis Categorical variables such as Frankel scores, Oswestry Disability Index (ODI) categories, SF-12 health status, and treatment outcomes are presented as descriptive categories. Visual Analog Scale (VAS) scores are presented as individual pre- and post-operative values per patient. Although no statistical test is presented within this table, a significant reduction in VAS scores was observed across all patients based on the Wilcoxon signed-rank test (p < 0.05).

Case	Age/sex	Site	Symptoms and duration	Imaging and diagnosis	Diagnosis (confirmatory)	Surgery	Treatment	Drug resistance/sensitive	Comorbidities	Pre- and post-OP VAS	Pre- and post-OP Frankel	ODI	SF-12	Outcome
1	51/F	Spine	Neck pain for six months	MRI and contrast MRI C5-C6 infective spondylodiscitis	Biopsy	CT-guided biopsy	Anti-tubercular therapy for 12 months (2+10)	Sensitive	Diabetes mellitus	8/10 to 2/10	E to E	Minimum disability	Average health	Treatment completed (clinico-radiological cure)
2	20/F	Spine	Lower back ache for 20 days	MRI and contrast MRI T11 tubercular spondylitis	Biopsy	CT-guided biopsy	Anti-tubercular therapy for 12 months (2+10)	Sensitive	Nil	7/10 to 2/10	E to E	Minimum disability	Good health	Treatment completed (clinico-radiological cure)
3	58/M	Spine	Radicular pain in the bilateral lower limbs for one month	MRI and contrast MRI D9-D10 discitis with cord compression	Biopsy	D9-D10 decompression and D8-D11 stabilisation	Anti-tubercular therapy for 12 months (2+10)	Sensitive	Nil	8/10 to 2/10	E to E	Moderate disability	Average health	Treatment completed (clinico-radiological cure)
4	44/M	Spine	Low back pain for three weeks. Bilateral lower limb radicular pain for three weeks	MRI and contrast MRI L1 vertebral burst fracture and infective spondylodiscitis (Figure 1)	Biopsy	T11-L3 posterior burst stabilization + T12 laminectomy + decompression	Anti-tubercular therapy for 12 months (2+10)	Sensitive	Retropositive	8/10 to 3/10	E to E	Moderate disability	Poor health	Treatment completed (clinico-radiological cure)
5	56/F	Spine	Lower back pain for six months. Bilateral lower limb radiculopathy for six months	MRI and contrast MRI L2—L3 infective spondylodiscitis with instability	Biopsy	L1-L5 posterior fusion + L2-L3 decompression and interbody fusion	Anti-tubercular therapy for 12 months (2+10)	Sensitive	Hypertension	9/10 to 3/10	E to E	Moderate disability	Average health	Treatment completed (clinico-radiological cure)
6	16/F	Spine	Lower back pain for 15 days. Fever for two days	MRI and contrast MRI L2-L3 infective spondylodiscitis (Figure 2)	Biopsy	Biopsy from the L2 vertebra through the transpedicular approach	Anti-tubercular therapy for 12 months (2+10)	Sensitive	Nil	6/10 to 2/10	E to E	Minimum disability	Good health	Treatment completed (clinico-radiological cure)
7	58/M	Spine	Lower back pain for one year	MRI, contrast MRI, and X-ray L4-L5 infective spondylodiscitis	Biopsy	L4-L5 decompression and L3-S1 stabilization	Anti-tubercular therapy for 12 months (2+10)	Sensitive	Diabetes mellitus and hypertension	8/10 to 2/10	E to E	Moderate disability	Average health	Treatment completed (clinico-radiological cure)
8	23/F	Spine	Lower back pain for three days	MRI and contrast MRI L3-L4 infective spondylodiscitis	Biopsy	CT-guided biopsy	Anti-tubercular therapy for 12 months (2+10)	Sensitive	Nil	7/10 to 2/10	E to E	Minimum disability	Good health	Treatment completed (clinico-radiological cure)
9	25/F	Spine	Lower back pain for two months. Bilateral lower limb radicular pain for two months	MRI and contrast MRI L4-L5 infective spondylodiscitis	Biopsy	L4-L5 posterior fusion and L3-S1 decompression	Anti-tubercular therapy for 12 months (2+10)	Sensitive	Nil	8/10 to 2/10	E to E	Minimum disability	Average health	Treatment completed (clinico-radiological cure)
10	18/F	Spine	Pain in the middle and lower back for one year. Bilateral lower limb radiculopathy for one year	MRI and contrast MRI L5-S1 infective spondylodiscitis	Biopsy	L5-S1 stabilization + fusion with cage placement	Anti-tubercular therapy for 12 months (2+10)	Sensitive	Nil	7/10 to 2/10	E to E	Minimum disability	Good health	Treatment completed (clinico-radiological cure)
11	72/M	Spine	Lower back pain for three months. Bilateral lower limb tingling sensation for 15 days	MRI and contrast MRI L5-S1 infective spondylodiscitis with severe instability pain (Figure 3)	Biopsy	L4-S1 posterior fusion + L5-S1 discectomy + L4-L5 decompression + L5-S1 TLIF	Anti-tubercular therapy for 12 months (2+10)	Sensitive	Diabetes mellitus and hypertension	8/10 to 4/10	E to E	Moderate disability	Average health	Treatment completed (clinico-radiological cure)
12	24/M	Spine	Lower back pain for five months	MRI and contrast MRI L5-S1 infective spondylodiscitis	Biopsy	L5-S1 posterior fusion and decompression	Anti-tubercular therapy for 12 months (2+10)	Sensitive	Nil	7/10 to 2/10	E to E	Minimum disability	Good health	Treatment completed (clinico-radiological cure)
13	32/M	Spine	Lower back pain for two weeks	MRI and contrast MRI L5- S1 infective spondylodiscitis	Biopsy	CT-guided biopsy	Anti-tubercular therapy for 12 months (2+10)	Sensitive	Nil	7/10 to 2/10	E to E	Minimum disability	Average health	Treatment completed (clinico-radiological cure)
14	24/F	Spine	1) Pain in lower back since 2 months	MRI and Contrast MRI Right Sacroilitis with Periarticular And Right Iliac Fossa Collection (Figure 4)	Biopsy	Under Ultrasound Guidance, 12 French Pigtail Drainage of Right Sacroilitis Periarticular Collection was done, Free Flow Pus noted and Catheter Secured	Anti - Tubercular Therapy for 12 Months (2+10)	Sensitive	Nil	8/10 to 4/10	E to E	Minimum Disability	Average Health	Treatment Completed (Clinico-Radiological Cure)

**Table 2:** Clinical, radiological, and treatment profile of patients with extraspinal tuberculosis Categorical variables such as functional scoring systems, SF-12 health status, and treatment outcomes are presented as descriptive categories. Visual Analog Scale (VAS) scores are presented as individual pre- and post-operative values per patient. Although no statistical test is presented within this table, a significant reduction in VAS scores was observed across all patients based on the Wilcoxon signed-rank test (p < 0.05).

Case	Age/sex	Site	Symptoms and duration	Diagnosis (imaging)	Diagnosis (confirmatory)	Surgery	Treatment	Drug resistance/sensitive	Comorbidities	Pre- and post VAS	SF 12	Scoring system used (pre- to post-op scoring)	Outcome
1	29/M	Knee joint	Pain and swelling in the right knee for six months. Pain in the left ankle for 20 days	MRI and contrast MRI right knee chronic synovitis (Figure 5)	Biopsy	Diagnostic arthroscopy + open synovectomy of the right knee	Anti-tubercular therapy for 12 months (2+10)	Sensitive	Nil	8/10 to 4/10	Average health	Knee Society Score - good to excellent	Treatment completed (clinico-radiological cure)
2	27/M	Sternal abscess with costochondral junction involvement	Swelling over the sternum for one week	MRI and contrast MRI sternal abscess with costochondral (1st and 2nd) junction involvement and bony erosion (Figure 6)	FNAC	Incision and drainage (Figure 7)	Anti-tubercular therapy for 12 months (2+10)	Sensitive	Nil	3/10 to 0/10	Good Health	No specific scoring used. Complete resolution of symptoms and radiological healing	Treatment completed (clinico-radiological cure)
3	26/F	Hip joint	Pain in the left hip for one month	MRI and contrast MRI left hip arthritis	Biopsy	Left hip excision debridement + arthrolysis + biopsy taken + skeletal traction applied	Anti-tubercular therapy for 12 months (2+10)	Sensitive	Nil	8/10 to 2/10	Average health	Modified Harris hip score - Fair to Excellent	Treatment completed (clinico-radiological cure)
4	18/F	Elbow joint	Pain in the right forearm for two years	MRI and contrast MRI right elbow synovitis	Biopsy	Right elbow synovectomy	Anti-tubercular therapy for 12 months (2+10)	Sensitive	Nil	7/10 to 3/10	Good health	Mayo Elbow Performance Score - fair to excellent	Treatment completed (clinico-radiological cure)
5	42/F	Wrist joint	Pain and swelling of the right wrist for two years	MRI and contrast MRI right wrist arthritis with synovial hypertrophy	Biopsy	Synovial biopsy	Anti-tubercular therapy for 12 months (2+10)	Sensitive	Nil	8/10 to 3/10	Good health	Quick Dash Score - poor to good	Treatment completed (clinico-radiological cure)
6	23/F	Shoulder joint	Pain in the right shoulder for three months	MRI and contrast MRI monoarthritis of the right shoulder	Biopsy	CT-guided biopsy	Ant-tubercular therapy for 12 months (2+10)	Sensitive	Nil	7/10 to 2/10	Good health	Constant Murley Shoulder Score - fair to excellent	Treatment completed (clinico-radiological cure)

**Figure 2:**
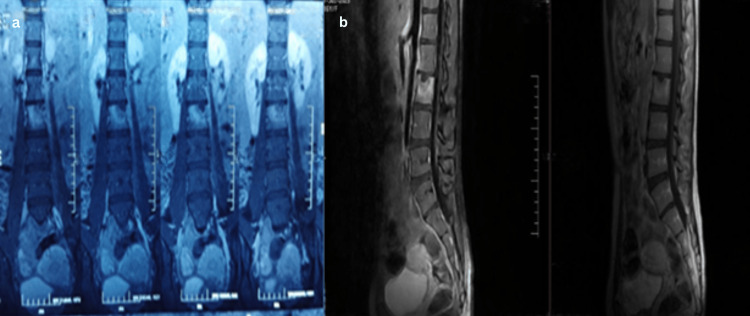
Spinal case 6 (a) Plain MRI of the lumbosacral region in the coronal view showing marrow intensity changes in both L1 and L2, extending to the cortical defect of the L2 superior endplate. (b) Contrast MRI in the sagittal view showing subtle post contrast enhancement in the anterior aspect of L1 inferior endplate and peripherally enhancing. Left paravertebral and prevertebral collection extending to the cortical defect of the L2 superior endplate. Data shown are representative MRI findings from a single patient case (spinal case 6). Original images obtained by the authors at Subbaiah Institute of Medical Sciences, Shivamogga.

**Figure 3:**
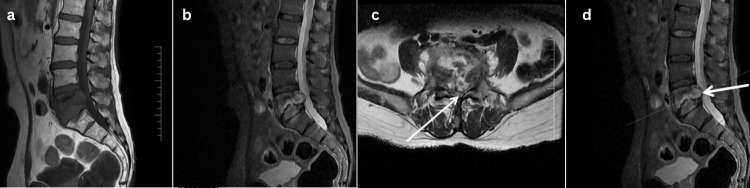
Spinal case 11 (a, b) MRI of the lumbar spine in the axial view and T2 sagittal views, respectively, showing severe T12-L1 disc retropulsion with compression of the conus medullaris and nerve roots, along with reactive inflammatory changes at this level. (c, d) Arrow mark indicates small epidural collection in the posterior aspect of the L5 vertebral body noted causing moderate canal stenosis. Data shown are representatives MRI findings from a single patient case (spinal case 11). Original images obtained by the authors at Subbaiah Institute of Medical Sciences, Shivamogga.

**Figure 4:**
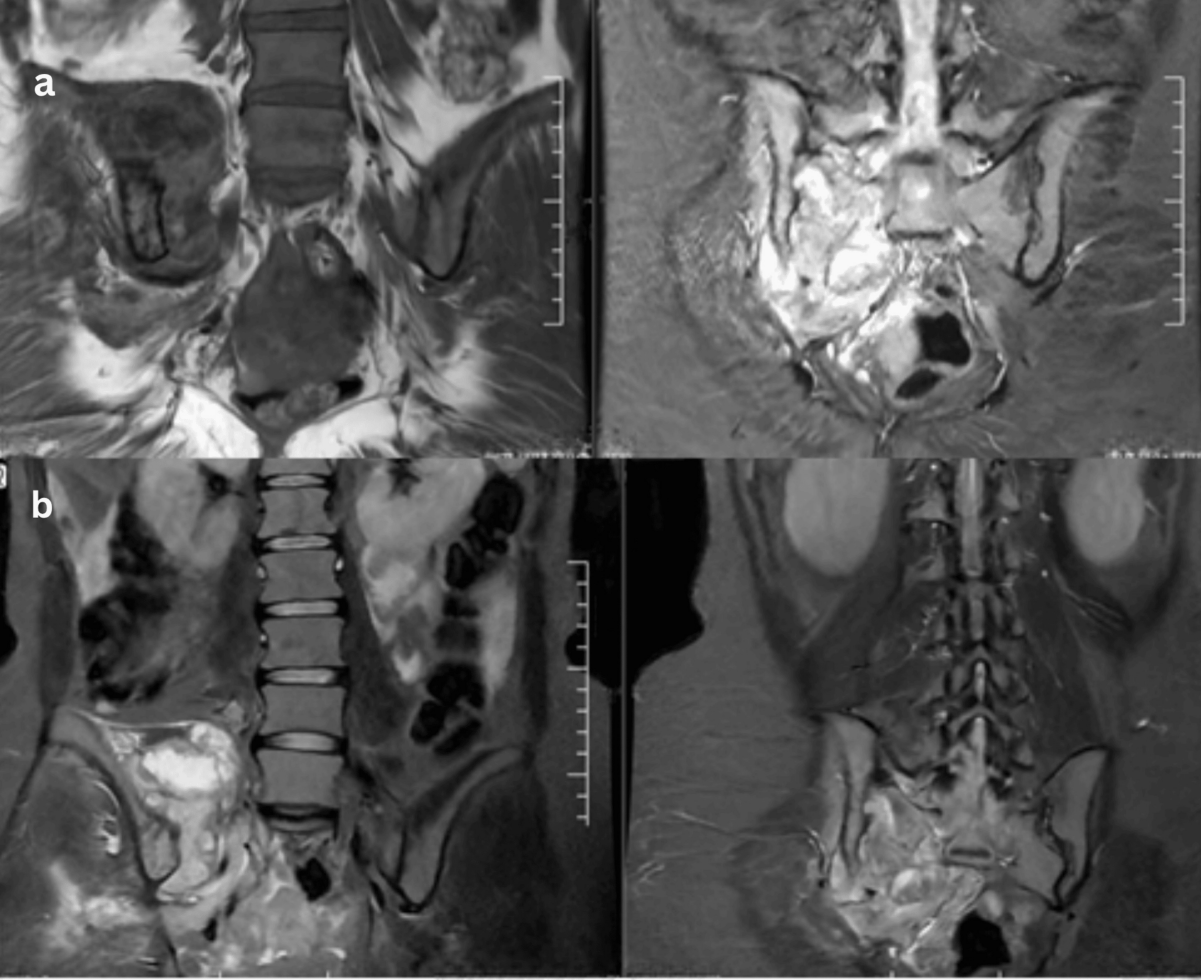
Spinal case 14 (a, b) Plain MRI and Contrast MRI, respectively, of the lumbosacral spine shows extensive bone marrow edema in the juxta articular regions of the right iliac bone and sacrum, further extending into the right sacral ala with evidence of bone abscess, significant synovial effusion, and multiple periarticular, peri-osseous, and intramuscular abscesses noted around the right sacroiliac joint. There is further extension of infection/abscesses into bilateral sacral neural foramina and lumbosacral spinal canal causing severe compression over cauda roots and severe spinal canal stenosis. Data shown are representative MRI findings from a single patient (spinal case 14). Original images obtained by the authors at Subbaiah Institute of Medical Sciences, Shivamogga.

**Figure 5:**
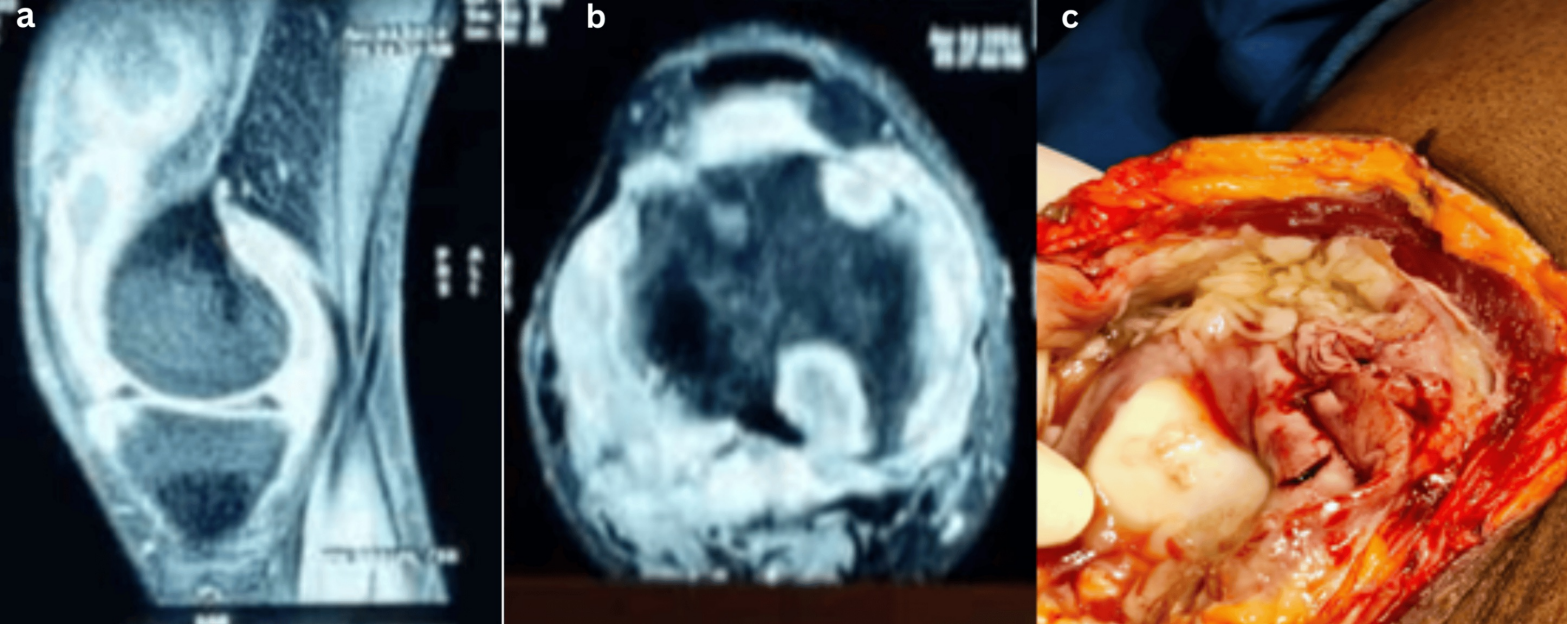
Extraspinal case 1: knee TB (a, b) MRI of the right knee joint in the sagittal and axial views, respectively, shows extensive synovitis with moderate knee joint effusion and multiple marginal erosions and patchy areas of bone marrow edema, suggestive of infective arthritis. (c) Intraoperative picture of the right knee joint showing caseous necrotic material within the joint cavity, surrounded by hypertrophied synovium with a thick, pale, and fibrillar appearance. The adjacent soft tissue and capsule appear hyperemic and edematous. Data shown are representative MRI and intraoperative findings from a single patient case (extraspinal case 1: knee TB). Original images obtained by the authors at Subbaiah Institute of Medical Sciences, Shivamogga.

Additional spinal TB presentations with their imaging features are shown in Figures 2-4. Spinal involvement was the most frequent presentation, observed in 14 of 20 patients (70%), while six patients (30%) had extra-spinal disease involving peripheral joints or unusual sites which included one case of knee (Figure 5), hip (one), shoulder (one), elbow (one), wrist (one), and sternum (one). Among the 14 spinal cases, the majority involved the lumbar region (six cases), with presentation of the youngest age being 16 years (Figure 2) and the oldest age being 72 (Figure 3), followed by the lumbosacral (four cases) and thoracic (two cases) regions. Single cases were observed in the cervical, thoracolumbar, and sacroiliac regions. MRI and intraoperative findings of tuberculous knee synovitis are shown in Figure 5.

The age range was 16-72 years, with a mean of 36.4 years. There was a slight female predominance, with 60% of the participants being female (12 females and eight males), resulting in a female-to-male ratio of 1.5:1. The duration of symptoms varied widely, from three days to two years with a mean duration of approximately 8.5 months, reflecting both acute presentations (such as abscesses or radicular pain) and indolent courses (as in elbow and wrist involvement). 

All patients underwent contrast-enhanced MRI, which demonstrated characteristic marrow, paraspinal, and soft-tissue changes consistent with skeletal TB. Histopathology showed necrotizing granulomatous inflammation compatible with TB, interpreted alongside NTEP-mandated clinicoradiological features. GeneXpert MTB/RIF detected no rifampicin resistance. No MDR-TB case was identified.

**Figure 6:**
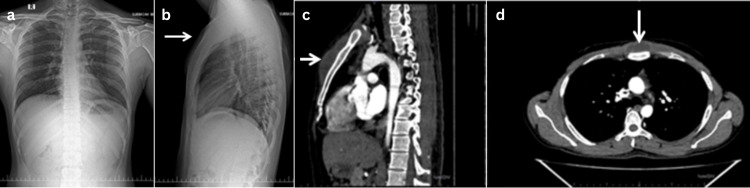
Extraspinal case 2: sternal TB (a, b) Chest X-ray posteroanterior (PA) view and lateral views, respectively, appears essentially normal. (b) The arrow in the lateral view indicates subtle soft tissue swelling. (c, d) High-resolution computed tomography (HRCT) of the thorax in sagittal and coronal views, respectively, arrow mark showing thick walled hypodense intramuscular collections with enhancing walls anterior to the manubrium, body of sternum, and left parasternal region, suggestive of an abscess. Data shown are representative radiographic findings from a single patient case (extraspinal case 2 - sternal TB). Original images obtained by the authors at Subbaiah Institute of Medical Sciences, Shivamogga.

**Figure 7:**
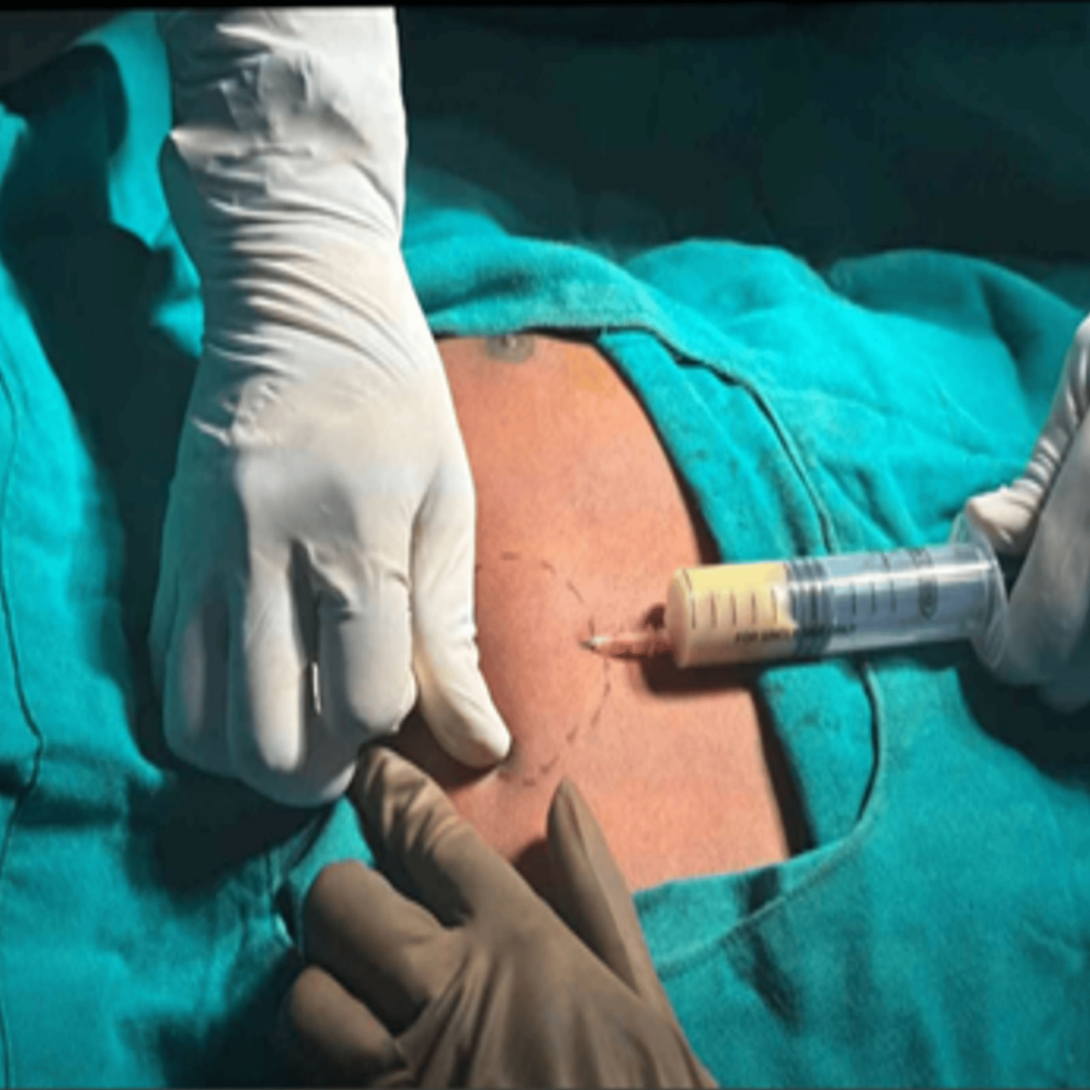
Extraspinal case 2: sternal TB Intraoperative aspiration of the thick and yellow purulent material from the pre-sternal abscess over the manubrium. Data shown represent intraoperative findings from a single patient case (extraspinal case 2: sternal TB). Original images obtained by the authors at Subbaiah Institute of Medical Sciences, Shivamogga.

Surgical intervention was required in patients with neurological deficits, mechanical instability, large abscesses, or advanced joint destruction. In the spine, the most common procedures were posterior decompression with stabilization and interbody fusion, while peripheral TB was addressed with synovectomy and debridement depending on disease severity. One patient with sternal TB (Figure 6) underwent incision and drainage, highlighting the wide clinical spectrum of skeletal TB. The radiological and surgical findings of sternal tuberculosis are demonstrated in Figures 6-7. 

In all cases, the standard NTEP treatment regimen protocol was followed. The treatment consisted of an intensive phase (IP) of two months, during which the patients received a fixed-dose combination of isoniazid (H), rifampicin (R), pyrazinamide (Z), and ethambutol (E). This was followed by a continuation phase (CP) of 10 months, during which patients received isoniazid (H), rifampicin (R), and ethambutol (E). All medications were administered as per weight-band dosing guidelines. 

The patients showed clinical improvement, which was also reflected in various scoring systems used for assessment. Functional outcomes were favourable. The mean pre-treatment VAS score for pain was 7.35 ± 1.23, which significantly decreased to 2.40 ± 0.94 following the intervention (n = 20). Pain improved significantly (median VAS 8 → 2, p < 0.001). Neurological status (Frankel grading) was normal (grade E) in all spinal cases preoperatively and remained unchanged postoperatively, with no deterioration observed. At final follow-up, quality of life (SF-12) scores improved notably: 1 = patient reported poor health, 10 = average, and 9 = good, paralleling improvements in ODI disability scores. 

Functional recovery of non-spinal cases was assessed with validated scoring systems: Knee Society Score: good to excellent, Modified Harris Hip Score: fair to excellent, Mayo Elbow Performance Score: fair to excellent, QuickDASH (wrist): poor to good, and Constant-Murley Shoulder Score: fair to excellent.

Comorbidities were present in four patients: two had both diabetes mellitus and hypertension, one had only diabetes, and one had only hypertension. One patient was HIV-positive, presenting with spinal TB. Treatment was successful in all patients, with each completing therapy and achieving clinico-radiological cure of TB.

## Discussion

Musculoskeletal TB remains a diagnostic challenge due to its variable presentation and frequent delay in recognition. Ozdemir et al. documented 31 musculoskeletal TB cases (3.2% of all TB cases) with a mean age of 44.2 years [21], whereas our study demonstrated a younger profile (mean age 36.4 years). Prasad et al. reported an almost equal gender distribution [22], while our series showed a female predominance (1.5:1), possibly reflecting differing healthcare access or exposure patterns in our population.

Spinal involvement constituted the majority of cases in our study, with lumbar predominance. By contrast, Pertuiset et al. observed thoracic and thoracolumbar involvement as the most common sites [2]. MRI findings in our spinal cases, such as marrow edema, disc space involvement, paravertebral collections, and associated soft-tissue changes, correspond with imaging characteristics reported by Pigrau-Serrallach and Rodríguez-Pardo [9].

Extraspinal TB in our cohort included the hip, knee, elbow, wrist, shoulder, and sternum. This anatomical diversity aligns with observations from McGuire et al. [14] and reflects the rare occurrence of sternal TB described by Khan et al. [23].

Diagnosis of skeletal TB requires careful clinicoradiological correlation due to low bacillary yield. Biopsy is recommended for patients with suspicious lesions, as emphasized by Colmenero et al. [7]. In our study, all cases were biopsy-confirmed, demonstrating necrotizing granulomatous inflammation or caseating granulomas. Fever was present in only one spinal case in our series, consistent with Colmenero et al., who noted that only about one-third of patients with spinal TB exhibit systemic fever [7]. Molecular tools that simultaneously detect *Mycobacterium tuberculosis* and rifampicin susceptibility have improved diagnostic confidence [7], although all our patients demonstrated drug sensitivity under NTEP protocols.

Treatment in skeletal TB should be tailored according to disease severity. Qiu et al. demonstrated that both anterior and posterior approaches for thoracic and lumbar TB offer comparable outcomes in terms of pain relief, neurological recovery, fusion rates, complications, operative time, and hospital stay [4]. In our series, all spinal surgeries were performed using the posterior approach with favorable outcomes.

Sternal TB, although rare, should be differentiated from malignancy and chronic infections. The presentation in our case-swelling with minimal systemic symptoms-was similar to that described by Khan et al. [23]. Shoulder TB requires management based on disease severity, and Kulkarni et al. demonstrated favorable outcomes using tailored protocols that range from arthroscopic to open debridement [24], which mirrors our management approach.

Management of peripheral joints followed established principles. Early hip involvement was managed conservatively, while advanced disease required surgical debridement, as recommended by Saraf et al. [12], similar to our case in which the hip was debrided due to joint destruction. Wrist TB commonly responds to ATT, as noted by Kotwal et al. [16], and our case showed a similar response. Delayed diagnosis remains an issue in peripheral disease, as described by Liao et al. for elbow TB [15], and supported by the long-standing knee disease reported by Soeroso et al., where synovectomy followed by one year of therapy achieved functional recovery without arthroplasty [25].

Watts et al. reported that while synovial analysis can confirm TB in a substantial proportion of skeletal cases, the remainder typically require biopsy demonstrating caseating granulomas [26]. This was consistent with our study, in which all diagnoses were confirmed by biopsy.

In a large series of vertebral osteomyelitis, Colmenero et al. identified underlying immunosuppression as a significant risk factor for tuberculous vertebral disease [8], which highlights the importance of considering spinal TB in immunocompromised individuals. Our study included one HIV-positive patient with spondylodiscitis. Despite the increased risk of extrapulmonary TB in HIV-infected individuals, the patient achieved full recovery with standard ATT and surgical management, highlighting the effectiveness of combined treatment when adherence is maintained.

In summary, early radiological assessment supported by tissue diagnosis, selective surgical intervention when required, and adherence to standardized NTEP-based therapy contributed to meaningful pain reduction and favorable functional recovery in our patients. The consistent treatment success observed highlights the value of program-based care and the need for sustained clinical suspicion to minimize diagnostic delay in skeletal TB.

Limitations**

This study was limited by its retrospective single-center design and a fixed sample size determined by the number of eligible cases treated during the study period. Follow-up was restricted to one year after treatment completion, which may not reflect long-term recurrence or functional outcomes, and the sample size limited subgroup comparisons between spinal and extraspinal disease.

## Conclusions

NTEP-guided composite diagnosis using MRI, histopathology, and cartridge-based nucleic acid amplification test (CBNAAT) enabled reliable identification of skeletal tuberculosis in this study. Standardized ATT with selective surgical intervention produced meaningful pain relief and functional improvement at one year after treatment completion. Early imaging, timely biopsy, and adherence to NTEP protocols remain essential to optimize recovery and prevent disability. Larger multicenter studies with longer follow-up are needed to confirm these findings.
