# Correction: Skeletal Tuberculosis: Clinical and Radiological Profile, Management, and Outcomes in a Retrospective Study

**DOI:** 10.7759/cureus.c391

**Published:** 2026-01-07

**Authors:** Vinay Kumar SI, Akash Hosthota, Vinutha D, Bharath HD, Harish S Pai

**Affiliations:** 1 Orthopedics, Subbaiah Institute of Medical Sciences, Shivamogga, IND; 2 Orthopedics and Spine Surgery, Subbaiah Institute of Medical Sciences, Shivamogga, IND; 3 School of Medicine, Subbaiah Institute of Medical Sciences, Shivamogga, IND

The legends of Tables 1 and 2 have been corrected to remove an incorrect description of the statistical test and p-value. Although no statistical test is presented within these tables, a significant reduction in VAS scores was observed across all patients based on the Wilcoxon signed-rank test (p < 0.05).

